# *Rhizobium* sp. IRBG74 Alters *Arabidopsis* Root Development by Affecting Auxin Signaling

**DOI:** 10.3389/fmicb.2017.02556

**Published:** 2018-01-04

**Authors:** Catherine Z. Zhao, Jian Huang, Prasad Gyaneshwar, Dazhong Zhao

**Affiliations:** ^1^Whitefish Bay High School, Whitefish Bay, WI, United States; ^2^Department of Biological Sciences, University of Wisconsin-Milwaukee, Milwaukee, WI, United States; ^3^College of Life Science, Shandong Normal University, Jinan, China

**Keywords:** *Arabidopsis*, auxin signaling, *Rhizobium*, RNA-seq, root development

## Abstract

*Rhizobium* sp. IRBG74 not only nodulates *Sesbania cannabina* but also can enhance rice growth; however, the underlying molecular mechanisms are not clear. Here, we show that *Rhizobium* sp. IRBG74 colonizes the roots of *Arabidopsis thaliana*, which leads to inhibition in the growth of main root but enhancement in the formation of lateral roots. The promotion of lateral root formation by *Rhizobium* sp. IRBG74 in the *fls2-1* mutant, which is insensitive to flagellin, is similar to the wild-type plant, while the auxin response deficient mutant *tir1-1* is significantly less sensitive to *Rhizobium* sp. IRBG74 than the wild type in terms of the inhibition of main root elongation and the promotion of lateral root formation. Further transcriptome analysis of *Arabidopsis* roots inoculated with *Rhizobium* sp. IRBG74 revealed differential expression of 50 and 211 genes at 24 and 48 h, respectively, and a majority of these genes are involved in auxin signaling. Consistent with the transcriptome analysis results, *Rhizobium* sp. IRBG74 treatment induces expression of the auxin responsive reporter *DR5:GUS* in roots. Our results suggest that in *Arabidopsis Rhizobium* sp. IRBG74 colonizes roots and promotes the lateral root formation likely through modulating auxin signaling. Our work provides insight into the molecular mechanisms of interactions between legume-nodulating rhizobia and non-legume plants.

## Introduction

Legume nodulating rhizobia are among the most effective plant growth-promoting rhizobacteria as these bacteria form nitrogen-fixing symbiotic associations with legumes which are highly efficient in supplying nitrogen to the host plants (Graham and Vance, 2003; Peoples et al., 2009; Gyaneshwar et al., 2011). The legume-rhizobia symbiosis involves a complex signal exchange between rhizobia and the legume host (Oldroyd and Downie, 2006; Stacey et al., 2006; Jones et al., 2007). Apart from forming nitrogen-fixing symbiosis with legumes, many rhizobial strains are known to form growth promoting associations with non-legumes, such as maize (*Zea mays*), wheat (*Triticum aestivum*), and rice (*Oryza sativa*) (Biswas et al., 2000a,b; Chaintreuil et al., 2000; Gutiérrez-Zamora and Martínez-Romero, 2001; Ladha and Reddy, 2003; Yanni and Dazzo, 2010; Mitra et al., 2016). It has been suggested that the ability of rhizobia to secrete plant growth-promoting hormones, especially indole acetic acid (IAA), might be important for plant growth promotion (Biswas et al., 2000b; Mishra et al., 2008).

To better understand the mechanisms underlying the interactions between legume nodulating rhizobia and non-legumes, we have chosen *Rhizobium* sp. IRBG74, which nodulates several species of *Sesbania*, as a model rhizobia (Cummings et al., 2009). In addition, *Rhizobium* sp. IRBG74 was shown to enhance the growth of wetland rice cultivars by the mechanisms other than nitrogen fixation (Biswas et al., 2000a,b). We recently showed that *Rhizobium* sp. IRBG74 colonizes rice tissues internally and that rhizobial lipopolysaccharide plays an important role in *Rhizobium* colonization (Mitra et al., 2016). In contrast to detailed knowledge of the mechanisms involved in rhizobial-legume interactions, there is significant lack of understanding about the molecular details of how bacterial endophytes recognize and enter their plant hosts as well as the mechanisms by which endophytes enhance plant growth.

*Arabidopsis* is a model species for plant genetics (Arabidopsis Genome Intiative, 2000; Zhao et al., 2001, 2002) and in recent years it has been utilized as a model system to elucidate plant microbiome and to determine mechanisms of plant interactions with beneficial microorganisms, especially the endophytes (Lebeis et al., 2015; Müller et al., 2016; Wintermans et al., 2016; Asari et al., 2017). Earlier studies showed that *Arabidopsis* can be colonized by *Azorhizobium caulinodans* ORS571, a rhizobial symbiont of aquatic legume *Sesbania rostrata* (Gough et al., 1997; Stone et al., 2001). A recent report has suggested that *Mesorhizobium loti* colonizes *Arabidopsis* roots and enhances plant growth likely through changes in plant auxin levels (Poitout et al., 2017). However, the mechanistic details of interactions between *Arabidopsis* and legume nodulating rhizobia has not yet been studied. The present study is focused on examining whether *Rhizobium* sp. IRBG74 can colonize *Arabidopsis* and the response of *Arabidopsis* to rhizobial inoculation at morphological and molecular levels.

## Materials and methods

### Bacterial strains, plant materials, and growth conditions

The *Rhizobium* sp. IRBG74 wild-type strain as well as strains marked with GUS (*Rhizobium* sp. IRBG74-GUS) and GFP (*Rhizobium* sp. IRBG74-GFP) (Mitra et al., 2016) reporters were maintained on LB agar plates. *Arabidopsis thaliana* Landsberg *erecta* (L*er*) and Columbia (Col-0), *fls2-1* (Gómez-Gómez and Boller, 2000) and *tir1-1* (Gray et al., 2001) mutants (Col-0), as well as the *DR5:GUS* transgenic line (Ulmasov et al., 1997) were used for studying colonization by *Rhizobium* sp. IRBG74. Seedlings were grown on the Murashige and Skooge (MS) medium in square Petri dishes under a 16-h light/8-h dark photoperiod regime at 22°C and 50% humidity.

### Colonization analysis of *Arabidopsis* roots inoculated by *Rhizobium* sp. IRBG74

The inoculation of *Arabidopsis* seedlings by *Rhizobium* sp. IRBG74 (wild type), *Rhizobium* sp. IRBG74-GUS and *Rhizobium* sp. IRBG74-GFP strains was carried out as described earlier (Mitra et al., 2016). Briefly, *Arabidopsis* seeds were surface-sterilized by soaking in 50% of household bleach for 10 min and then in 70% ethanol for further 10 min. Sterilized seeds were then planted on Petri dishes containing MS salt and stratified for 3 days at 4°C and then germinated at 22°C in a growth chamber. Roots of 4-day old seedlings were inoculated with LB grown bacterial strains (0.1 O.D.) or plain LB as control. Seedlings were then transferred to square Petri dishes with the MS medium and incubated in a growth chamber at 22°C. Three biological repeats were performed with 10 seedlings per treatment.

The colonization of *Arabidopsis* roots was examined at 2 and 7 days after inoculation using histochemical GUS staining (Liu et al., 2010; Huang et al., 2016d) and fluorescence microscopy (Huang et al., 2016b,c, 2017) as described in our previous studies. The seedlings were removed from the plates and washed three times with sterile water and then used for GUS staining and GFP fluorescence analyses. The images of GUS stained roots were photographed by an Olympus SZX7 dissection microscope equipped with the Olympus DP 70 digital camera (Olympus, Center Valley, PA, USA). For confocal microscopy analysis, root samples were observed with a Leica TCS SP2 laser scanning confocal microscope using a 63×/1.4 water immersion objective lens. A 488-nm laser was used to excite GFP and FM4-64. The emission was captured using PMTs set at 505–530, 500–550, and 644–719 nm, respectively.

### Analysis of *Arabidopsis* root development after treatment with *Rhizobium* sp. IRBG74

As described above, *Arabidopsis* wild-type, *fls2-1* and *tir1-1* mutant seedling were inoculated with the LB grown *Rhizobium* sp. IRBG74 (wild type) strain and the plain LB, respectively. The effect of bacterial inoculation on root development was determined by measuring the length of primary root and the numbers of lateral roots 7 days after inoculation. Three biological repeats were performed with 10 seedlings per treatment. Data were analyzed for statistical significance by *t*-test.

### Transcriptome analysis of *Arabidopsis* root development in response to treatment with *Rhizobium* sp. IRBG74

To examine how the *Arabidopsis* root development responds to the *Rhizobium* sp. IRBG74 treatment at the molecular level, we performed RNA-seq experiments as described in our previous studies (Huang et al., 2016d). One hundred seedlings were inoculated with the LB grown *Rhizobium* sp. IRBG74 (wild type) strain and the plain LB (control). Roots were collected at 24 and 48 h after inoculation and then were used for RNA extraction by RNeasy Plant Mini Kit (Qiagen). On-column DNase digestion was carried out using the RNase-free DNase (Qiagen). RNA amounts were determined by the Qubit 3.0 Fluorimeter (Fisher scientific) and 500 ng of total RNA was used for preparing RNA-seq library. RNA-seq libraries were constructed using the Illumina's TruSeq Stranded Total RNA with Ribo-Zero Plant kit. Three biological repeats were performed; therefore, totally 12 libraries were sequenced (single-end sequencing, 1 × 100 bp) in the Biotechnology Center of University of Wisconsin-Madison on a HiSeq2500 (Illumina) using a TruSeq SBS sequencing kit version 3 (Illumina) and processed with Casava 1.8.2. The average reads for each library are 27.2 M.

RNA-seq data evaluation and pre-processing were performed by the CyVerse Discovery Environment (Goff et al., 2011; Merchant et al., 2016). The quality of RNA-seq reads was evaluated by FastQC 0.10.1. Adapter sequences were removed by Scythe-adapter-trimming. Low quality sequences were trimmed and filtered by Sickle-quality-based-trimming. The read-quality of the cleaned reads was re-evaluated by FastQC 0.10.1. RNA-Seq reads were aligned to the *Arabidopsis* genome using TopHat2-SE. Cufflinks2 was used to assemble transcripts from the RNA-seq data. Cuffmerge2 was used to merge all Cufflinks transcripts into a single transcriptome annotation file. Cuffdiff2.2.1a was used to compare differentially expressed genes. Genes with *p*-values < 0.05 and log2 fold changes more than 1 or less than−1 were selected as differentially expressed genes and were further analyzed. GO annotation search and functional categorization were performed from TAIR (https://www.arabidopsis.org).

Genes were clustered using the Database for Annotation, Visualization and Integrated Discovery (DAVID) (http://david.abcc.ncifcrf.gov/) (Huang et al., 2007, 2009, 2016d). The files of output DAVID chart records were used to generate enrichment map (Merico et al., 2011). Only gene-sets passing conservative significance thresholds (*p*-value < 0.001, False Discovery Rate (FDR) < 2%, the overlap coefficient = 0.5) were selected to present in the Enrichment Map, which results in 106 significantly enriched gene-sets. Heat-maps were generated by the Enrichment Map software. The complete datasets in this study are available in the NCBI GEO database under accession numbers of GSM2863566, GSM2863567, GSM2863568, GSM2863569, GSM2863570, GSM2863571, GSM2863572, GSM2863573, GSM2863574, GSM2863575, GSM2863576, and GSM2863577.

### Characterization of auxin response in *Arabidopsis* roots inoculated with *Rhizobium* sp. IRBG74

Seedlings of the *DR5:GUS* transgenic line were inoculated with the LB grown *Rhizobium* sp. IRBG74 (wild type) strain and the plain LB (control). As described in our previous studies (Liu et al., 2010; Huang et al., 2016d), changes in GUS expression were determined using histochemical GUS staining and quantification of GUS activity using a Synergy HT multi-mode microplate reader at 360 nm (excitation) and 460 nm (emission). The concentration of protein was measured using the Bradford method and GUS activity was calculated as nM 4-MU/min/mg protein.

## Results and discussion

### *Rhizobium* sp. IRBG74 can colonize *Arabidopsis* roots

**Figure 1:**
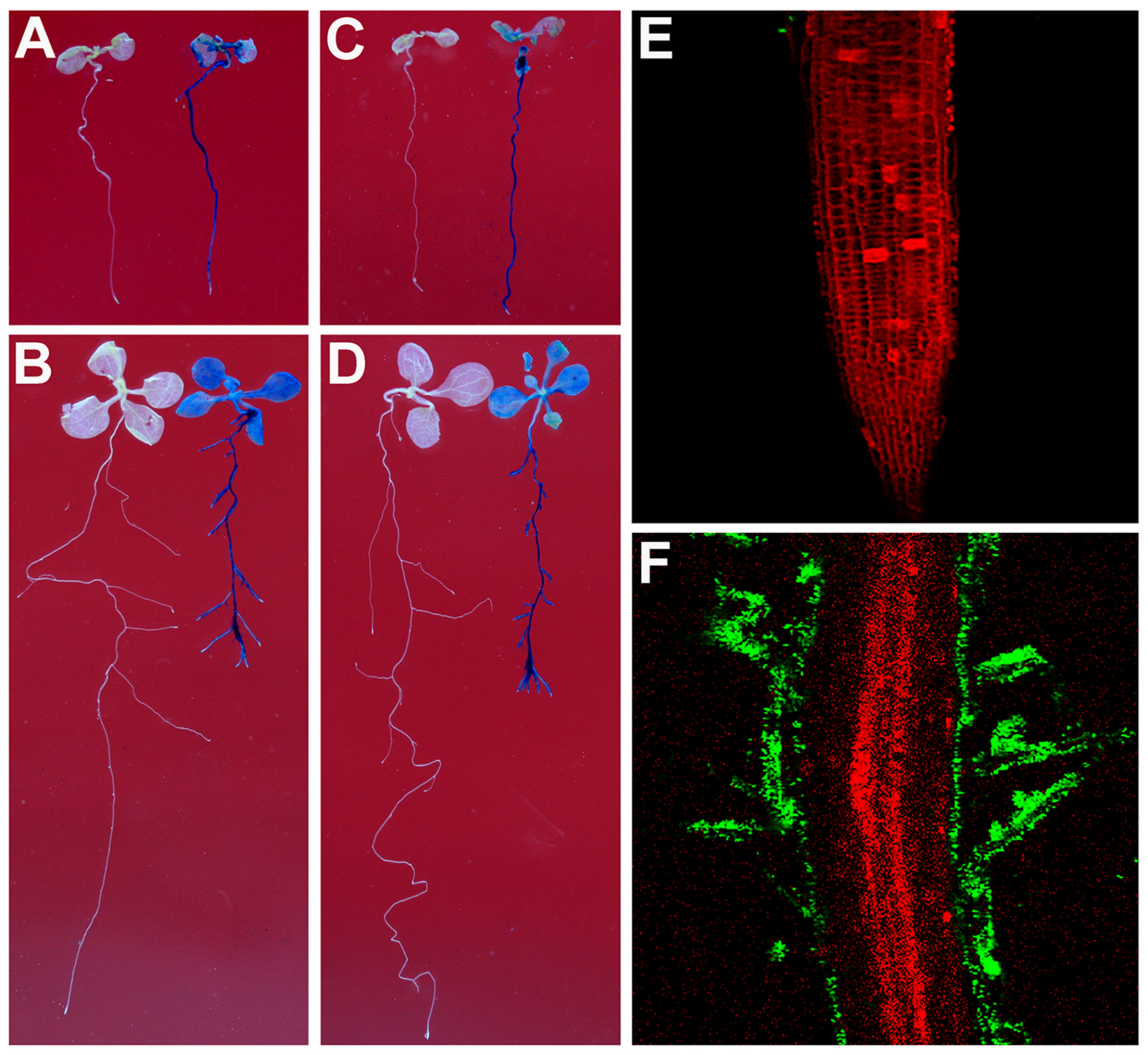
*Rhizobium* sp. IRBG74 can colonize *Arabidopsis* Root. **(A,B)** Columbia (Col-0) 4-day seedlings without (left) and with (right) *Rhizobium* sp. IRBG74-GUS treatment for 2 **(A)** and 7 **(B)** days. **(C,D)** Landsberg *erecta* (L*er*) 4-day seedlings without (right) and with (right) *Rhizobium* sp. IRBG74-GUS treatment for 2 **(C)** and 7 **(D)** days. **(E)** No GFP signal was found in the root tip of Col-0 4-day seedlings treated with *Rhizobium* sp. IRBG74-GFP for 2 days. **(F)** GFP signal was detected in root hairs of Col-0 4-day seedlings treated with *Rhizobium* sp. IRBG74-GFP for 2 days.

To test whether *Rhizobium* sp. IRBG74 can colonize the *Arabidopsis* root, we inoculated *Arabidopsis* (Col-0 and L*er*) roots with strains of *Rhizobium* sp. IRBG74-GUS and IRBG74-GFP which were utilized to study colonization of *Sesbania cannabina* and rice before (Mitra et al., 2016). From 30 Col-0 seedlings (3 repeats, each repeat contains 10 seedlings) treated by *Rhizobium* sp. IRBG74-GUS, GUS signals were observed throughout the main roots of all seedlings by 2 *D*ays *A*fter *I*noculation (DAI) (Figure 1A and Supplementary Figure 1) and on both the main root and lateral roots of all seedlings at 7 DAI (Figure 1B). A similar result was obtained from *Rhizobium* sp. IRBG74-GUS treated L*er* seedlings (Figure 1C,D). Confocal microscopy further demonstrated that although no GFP signal was observed in the root tips of 30 examined seedlings (3 repeats, each repeat contains 10 seedlings) treated by *Rhizobium* sp. IRBG74-GFP at 2 DAI (Figure 1E), the GFP expression was detected in root hairs (the surface of main root) at 7 DAI (Figure 1F), which agrees with the results from the GUS staining (Figures 1A,C). The fluorescent bacteria were found mainly on the root surface. These results suggest that *Rhizobium* sp. IRBG74 can colonize *Arabidopsis* roots epiphytically under the studied conditions.

### *Rhizobium* sp. IRBG74 promotes lateral root formation in *Arabidopsis*

**Figure 2:**
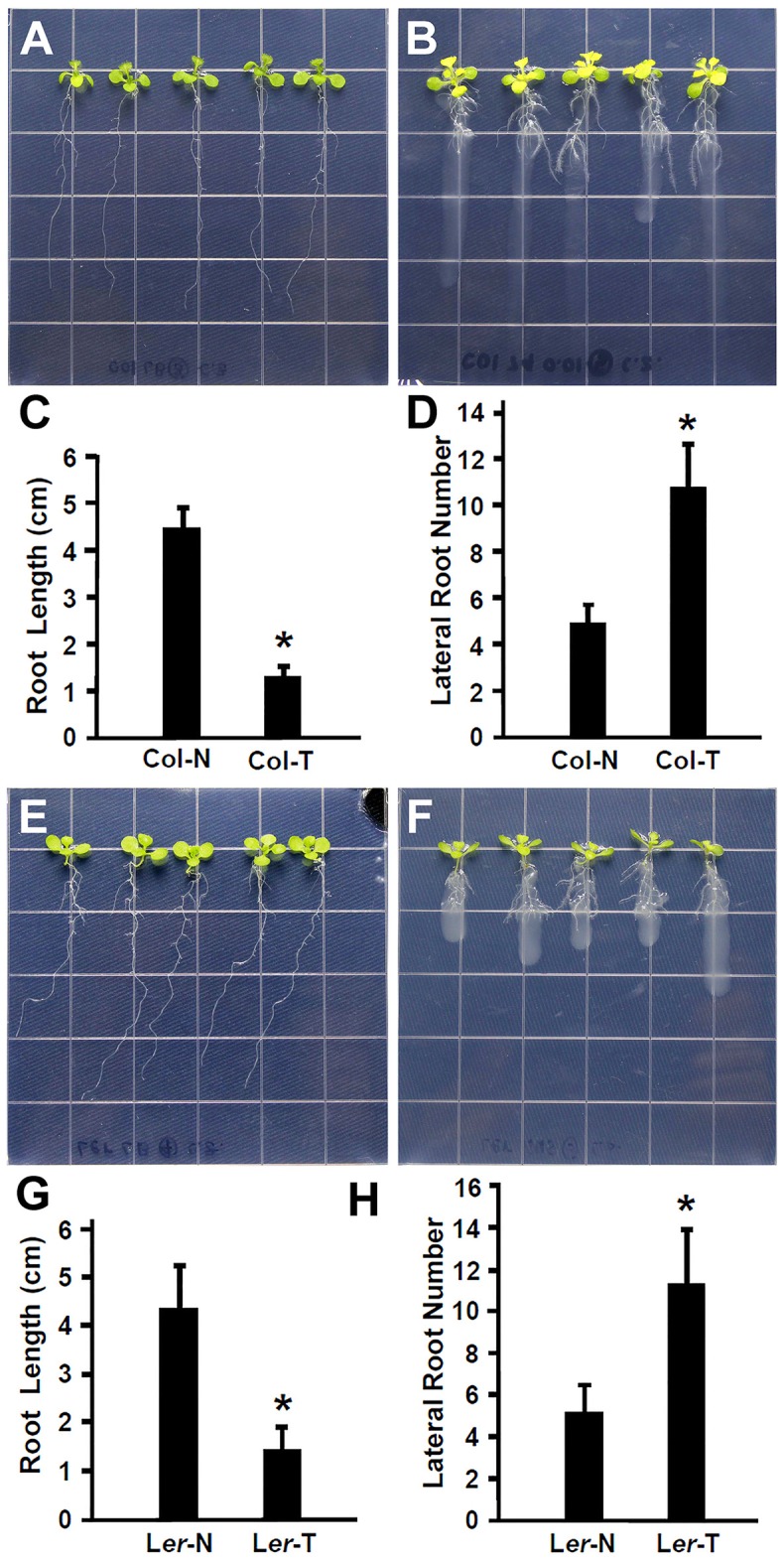
*Rhizobium* sp. IRBG74 promotes lateral root formation but inhibits the primary root growth in *Arabidopsis*. **(A,B)** Columbia (Col-0) 4-day seedlings without **(A)** and with **(B)** *Rhizobium* sp. IRBG74 treatment for 7 days. **(C)** Primary root length of Col-0 4-day seedlings without (Col-N) and with (Col-T) *Rhizobium* sp. IRBG74 treatment for 7 days. **(D)** Lateral root numbers of Col-0 4-day seedlings without (Col-N) and with (Col-T) *Rhizobium* sp. IRBG74 treatment for 7 days. **(E,F)** Landsberg *erecta* (L*er*) 4-day seedlings without **(E)** and with **(F)** *Rhizobium* sp. IRBG74 treatment. **(G)** Primary root length of L*er* 4-day seedlings without (L*er*-N) and with (L*er*-T) *Rhizobium* sp. IRBG74 treatment for 7 days. **(H)** Lateral root numbers of L*er* 4-day seedlings without (L*er*-N) and with (L*er*-T) *Rhizobium* sp. IRBG74 treatment for 7 days. ^*^Indicates the difference is significant (*P* < 0.01).

Recent studies have suggested that plant beneficial bacteria enhance the formation of lateral roots in host plants, which in turn allow more surface area for bacteria to colonize (Gyaneshwar et al., 2002; James et al., 2002). As shown in Figures 1A–D, *Rhizobium* sp. IRBG74 promoted the lateral root formation but inhibited the main root elongation. To further study this effect, the length of the main root and the number of lateral roots were quantified. Inoculation of 30 (3 repeats, each repeat contains 10 seedlings) *Arabidopsis* seedlings with *Rhizobium* sp. IRBG74 resulted in significant reduction in growth of the main root and significant enhancement in the number of latera roots at 7 DAI in both Col-0 (Figures 2A–D) and L*er* ecotypes (Figures 2E–H). These results are consistent with earlier reports showing the enhancement of *Arabidopsis* root growth by *Baccillus amyloliquefaciens* SQR9 (Chen et al., 2017) and the increase in rice root growth by *Rhizobium* sp. IRBG74 (Mitra et al., 2016). Additionally, earlier studies have shown that the natural openings created as a result of emergence of lateral roots from the main root could allow point of entry for plant beneficial bacteria through “crack entry,” especially in non-legumes (Gyaneshwar et al., 2002; James et al., 2002). *Rhizobium* sp. IRBG74 is known to colonize its plant-hosts through “crack entry” (Mitra et al., 2016). Our results suggests that *Rhizobium* sp. IRBG74 likely induces the formation of lateral roots which in turn might provide additional openings for the bacteria to invade the internal tissues.

### The enhancement of lateral root formation is not altered in *fls2-1* seedlings treated by *Rhizobium* sp. IRBG74

**Figure 3:**
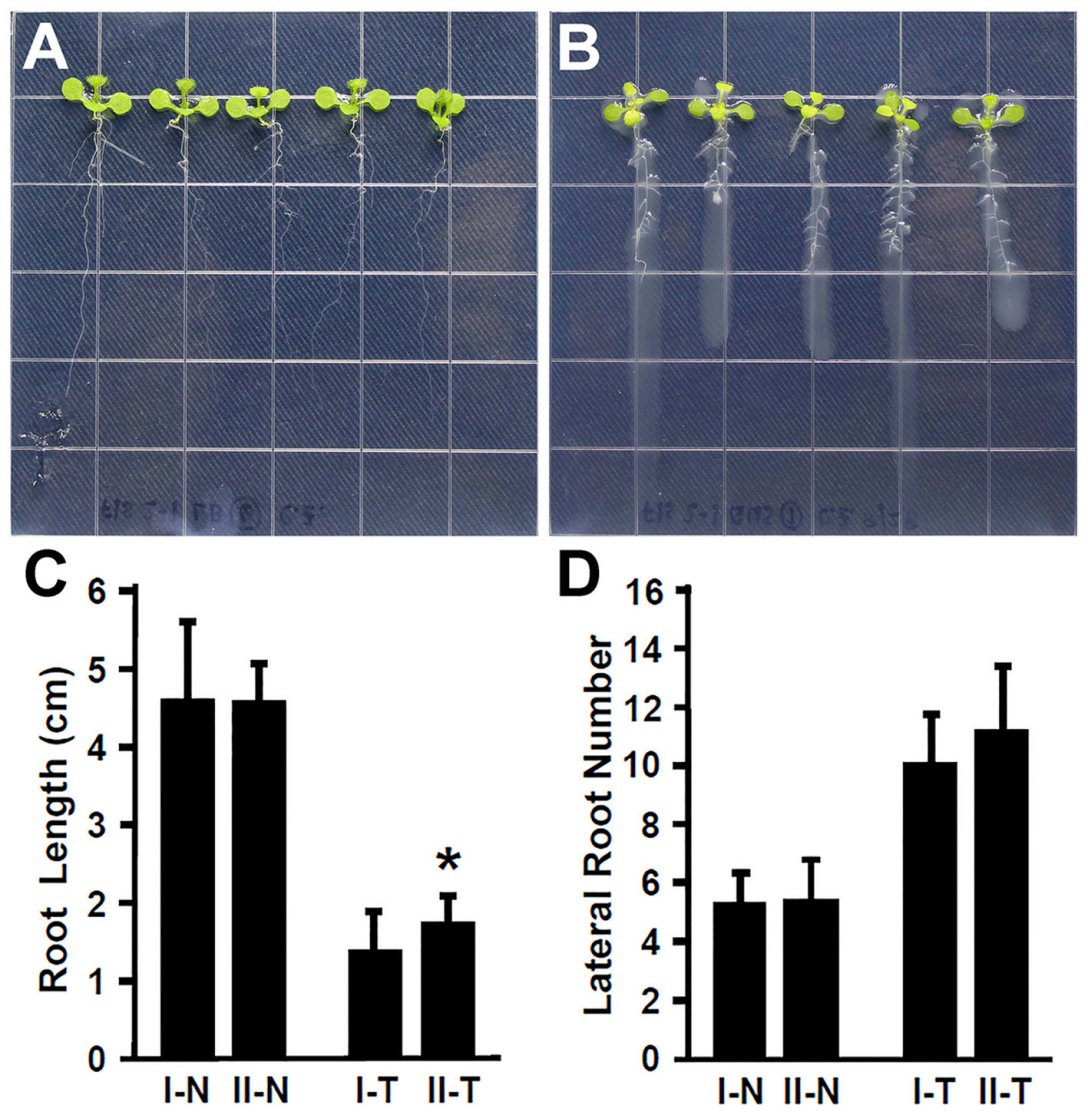
The enhancement of lateral root formation is not altered in *fls2-1* seedlings treated by *Rhizobium* sp. IRBG74. **(A,B)** *fls2-1* mutant 4-day seedlings without **(A)** and with **(B)** *Rhizobium* sp. IRBG74 treatment for 7 days. **(C)** Primary root length of Col-0 and *fls2-1* 4-day seedlings without and with *Rhizobium* sp. IRBG74 treatment for 7 days. **(D)** Lateral root numbers of Col-0 and *fls2-1* 4-day seedlings without and with *Rhizobium* sp. IRBG74 treatment for 7 days. I-N, Col-0 without *Rhizobium* sp. IRBG74 treatment; II-N, *fls2-1* without *Rhizobium* sp. IRBG74 treatment; I-T, Col-0 with *Rhizobium* sp. IRBG74 treatment; and II-T, *fls2-1* with *Rhizobium* sp. IRBG74 treatment. ^*^Indicates the difference is significant (*P* < 0.01).

Plant recognize the beneficial and pathogenic microorganisms through the Microbe-Associated Molecular Patterns (MAMPs) (Dangl and Jones, 2001). Bacterial flagellin is one of the MAMPs that triggers plant defense response upon its recognition by the FLAGELLIN SENSITIVE 2 (FLS2) receptor (Gómez-Gómez and Boller, 2000; Zipfel et al., 2004). The FLS2 receptor in *Arabidopsis* recognizes bacterial flagella and induces the plant defense response (Zipfel et al., 2004; Schwessinger and Ronald, 2012). It has been suggested that the lack of flagella might enhance the endophytic colonization (Capdevila et al., 2004; Iniguez et al., 2005). In contrast, rice colonization by growth promoting *Azoarcus* BH72 requires bacterial flagella (Buschart et al., 2012). To determine whether the flagellin recognition by *Arabidopsis* plays a role in colonization by *Rhizobium* sp. IRBG74, 30 (3 repeats, each repeat contains 10 seedlings) *fls2-1* mutant seedlings (Gómez-Gómez and Boller, 2000) were inoculated and changes in root development were determined. Similar to the wild type, *Rhizobium* sp. IRBG74 inhibits the elongation of main roots of *fls2-1* mutant seedlings; however the inhibition efficacy is lower (Figures 3A–C). In addition, *Rhizobium* sp. IRBG74 showed a similar effect in terms of enhancing lateral root formation in wild-type and *fls2-1* seedlings (Figures 3A,B,D). Our results suggest that flagellin recognition is unlikely involved in the ability of *Rhizobium* sp. IRBG74 to colonize *Arabidopsis* roots.

### The *tir1-1* mutant is insensitive to the treatment with *Rhizobium* sp. IRBG74

**Figure 4:**
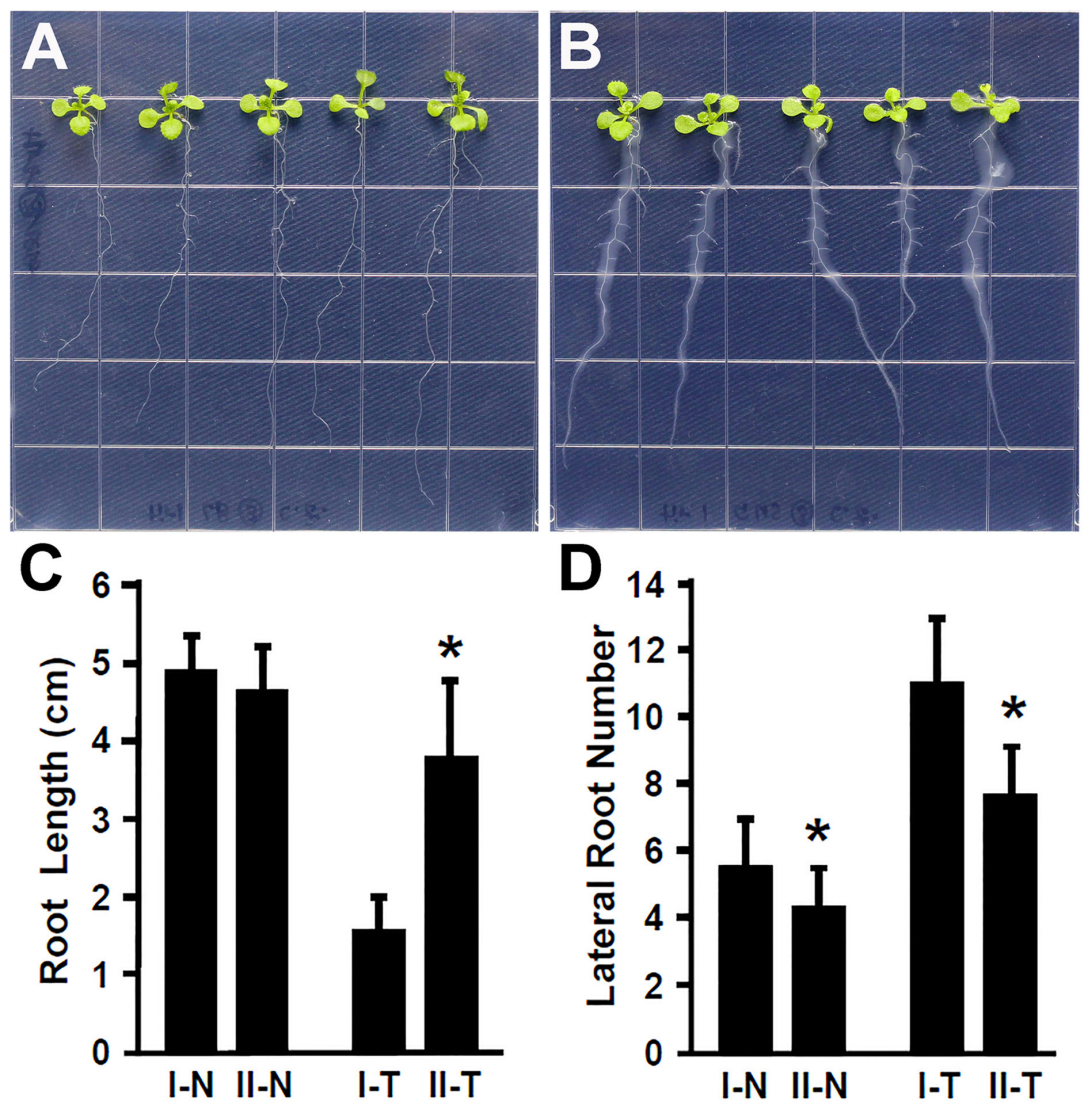
The *tir1-1* mutant is insensitive to *Rhizobium* sp. IRBG74 treatment. **(A,B)** *tir1-1* mutant 4-day seedlings without **(A)** and with **(B)** *Rhizobium* sp. IRBG74 treatment for 7 days. **(C)** Primary root length of Col-0 and *tir1-1* 4-day seedlings without and with *Rhizobium* sp. IRBG74 treatment for 7 days. **(D)** Lateral root numbers of Col-0 and *tir1-1* 4-day seedlings without and with *Rhizobium* sp. IRBG74 treatment for 7 days. I-N, Col-0 without *Rhizobium* sp. IRBG74 treatment; II-N, *tir1-1* without *Rhizobium* sp. IRBG74 treatment; I-T, Col-0 with *Rhizobium* sp. IRBG74 treatment; and II-T, *tir1-1* with *Rhizobium* sp. IRBG74 treatment. ^*^Indicates the difference is significant (*P* < 0.01).

Plant hormone auxin plays an important role in lateral root development (Fukaki et al., 2007; Gutierrez et al., 2009; Huang et al., 2016a). Our results showed that inoculation with *Rhizobium* sp. IRBG74 promoted the lateral root formation, suggesting that auxin is likely involved in the response of *Arabidopsis* to *Rhizobium* sp. IRBG74. To further explore this idea, the *Arabidopsis tir1-1* mutant which is defective in auxin perception (Gray et al., 2001; Dharmasiri et al., 2005; Kepinski and Leyser, 2005; Tan et al., 2007) was inoculated with *Rhizobium* sp. IRBG74 and its effect on root development was examined. In contrast to wild-type seedlings that showed a strong inhibition of main root elongation (Figure 2), the *tir1-1* mutant seedlings (30 total, 3 repeats) exhibited a significantly less reduction in main root growth (Figures 4A–C). The *tir1-1* mutant seedlings produced a less number of lateral roots than the wild type without *Rhizobium* sp. IRBG74 treatment; however, with the *Rhizobium* sp. IRBG74 treatment, the number of lateral roots in wild-type seedlings increased 98.19%, but the number of lateral roots in *tir1-1* seedlings increased 76.94% (Figures 4A,B,D). These results indicate that the *tir1-1* mutant is less sensitive than the wild type regarding the inhibition of main root elongation and promotion of lateral root formation. Auxin synthesized by plant beneficial bacteria is postulated to be a major mechanism by which these bacteria promote plant growth (Bloemberg and Lugtenberg, 2001; Lugtenberg and Kamilova, 2009). Our results suggest that *Rhizobium* sp. IRBG74 affects the main root growth and lateral root formation possibly by changing auxin signaling.

### *Rhizobium* sp. IRBG74 leads to differential expression of genes involved in auxin signaling, cell wall and cell membrane integrity, and transport during root development

To examine the molecular mechanisms by which *Rhizobium* sp. IRBG74 affects root development, we performed RNA-seq analysis using *Arabidopsis* seedling treated by *Rhizobium* sp. IRBG74. Roots of 4-day seedlings that were un-inoculated and inoculated with *Rhizobium* sp. IRBG74 for 24 and 48 h were sampled. Each sample contained 100 roots. Three biological repeats were performed; therefore, totally 12 libraries were constructed and sequenced. Analysis of transcriptome changes showed that 50 and 211 genes were differentially expressed at 24 and 48 h (Supplementary Tables 1, 2), respectively. Examination of these genes using Gene Ontology (GO) cellular component analysis demonstrated that proteins encoded by these genes may function in the extracellular region (13.884%), nucleus (10.896%), the plasma membrane (10.193%), and cell wall (6.854%, Supplementary Figure 2). In addition, GO molecular function analysis showed that the coding proteins have transferase (10.385%), hydrolase (8.462%), transporter (7.885%), protein binding (7.5%), kinase (5.769%), and transcription factor (5%) activities (Supplementary Figure 3). Moreover, GO biological process analysis further found that these genes are possibly involved in responses to biotic and abiotic stimulus/stress (19.423%), development (7.308%), transport (6.346%), signal transduction (5.577%), and gene transcription (4.712%, Supplementary Figure 4).

**Figure 5:**
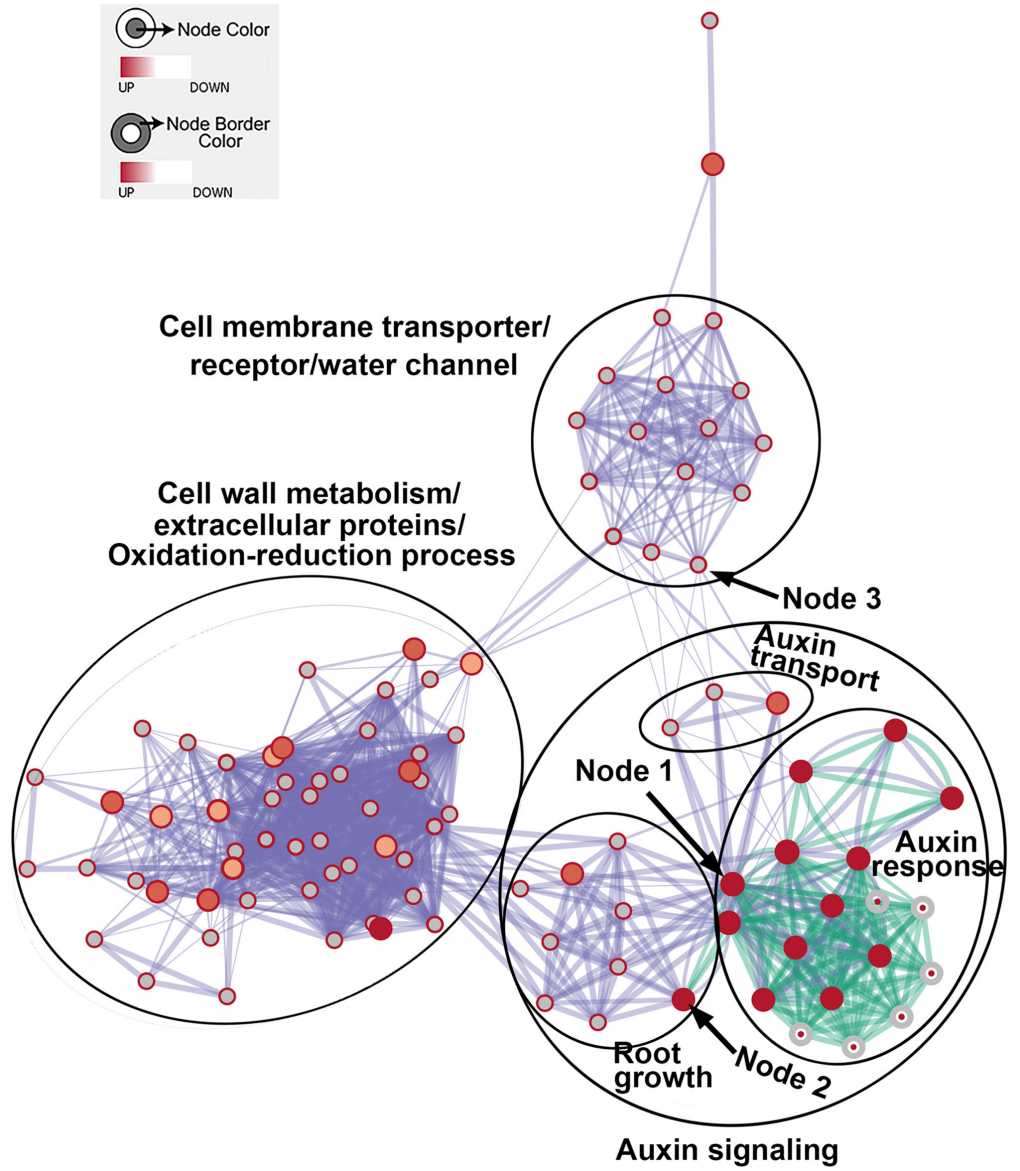
The enrichment map showing differentially expressed gene-sets in roots treated by *Rhizobium* sp. IRBG74 for 24 and 48 h. The map displays enriched gene-sets in untreated and treated roots at 24 and 48 h. The node center represents gene-sets at 24 h, whereas the node border represents gene-sets at 48 h. Gene-sets at 24 h are connected by green lines, while gene-sets at 48 h are linked by light blue lines. The node size represents the gene-set size and line thickness indicates the degree of overlap between two gene-sets. Clusters of gene-sets were manually circled and labeled to demonstrate biological and molecular functions. Node 1 represents auxin response genes, node 2 contains genes with functions in root growth, and node 3 represents cell membrane transporter/receptor/water channel genes.

We then generated enriched GO gene-sets using the Database for Annotation, Visualization and Integrated Discovery (DAVID) (Huang et al., 2007, 2009, 2016d; Merico et al., 2011). The enrichment map contains 106 significantly enriched gene-sets (Figure 5). Enriched gene-sets from the 24-h treatment (indicated by the node center) were connected by green lines, while gene-sets from the 48-h treatment (indicated by the node border) are linked by light blue lines.

**Figure 6:**
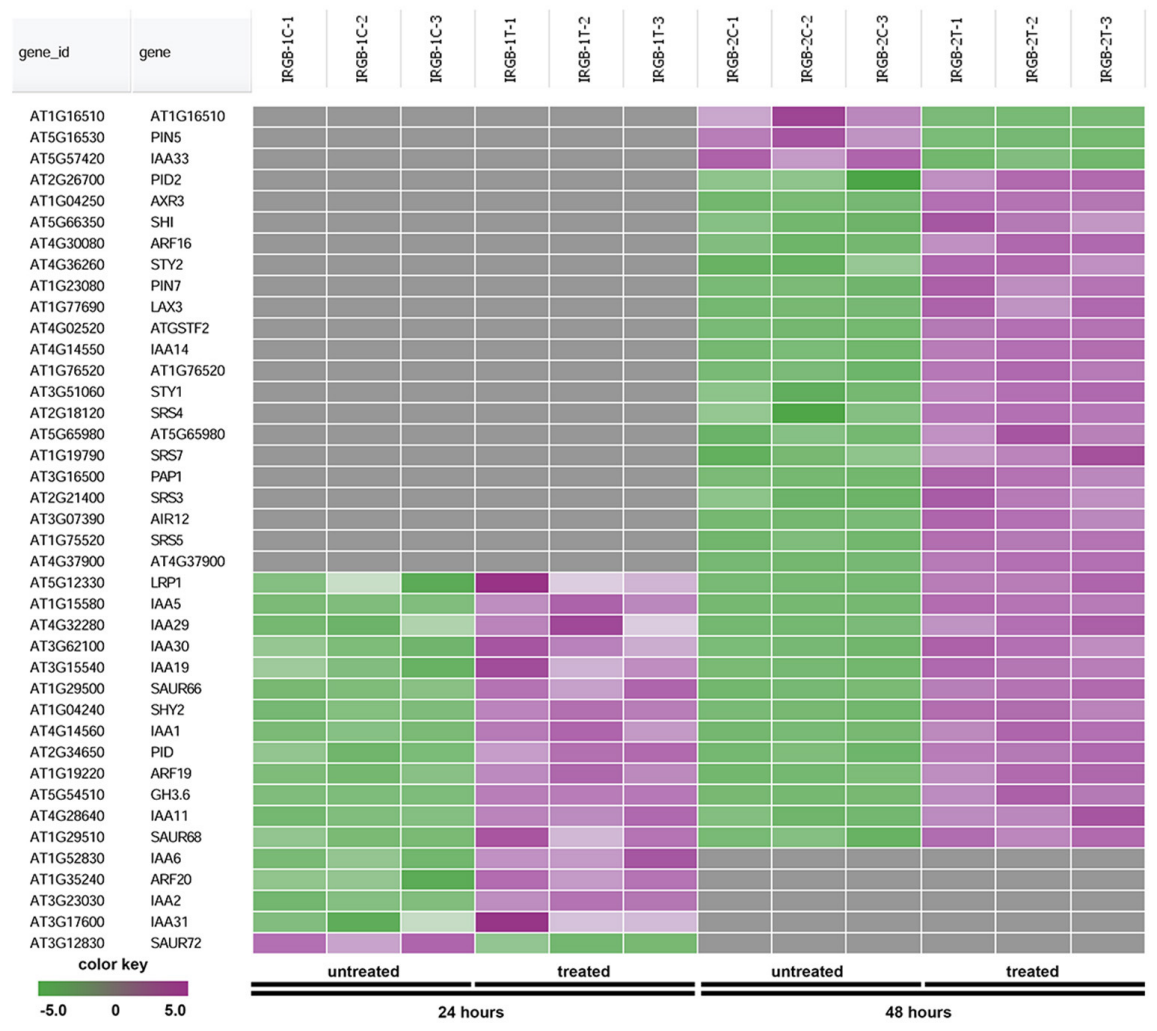
Heatmap showing expression changes of representative genes related to auxin signaling. The auxin signaling subgroup in the node 1 gene-set showing in Figure 5 contains a total of 40 genes. Green color: down-regulated; Purple color: up-regulated; and Gray color: no significant expression change.

In the enrichment map, three distinct groups represent auxin signaling, cell wall metabolism/extracellular protein/ oxidation-reduction process, and cell membrane reporter/transporter/water channel (Figure 5). The mainly overlapped gene-sets between genes expressed at 24 and 48 h are clustered into the subgroup of auxin signaling. The heatmap generated from the node 1 showed 40 auxin response genes, such as *IAA* (*IAA1, IAA2, IAA3*/*SHY2, IAA5, IAA6, IAA11, IAA19, IAA29, IAA30*, and *IAA31*), *SAUR* (*SAUR66* and *SAUR68*), *ARF* (*ARF19* and *ARF20*), *PIN* (*PIN5* and *PIN7*), *PID*, and *GH3.6* genes (Figure 6 and Supplementary Table 3). More auxin signaling genes were expressed at 48 h, including auxin transport genes.

**Figure 7:**
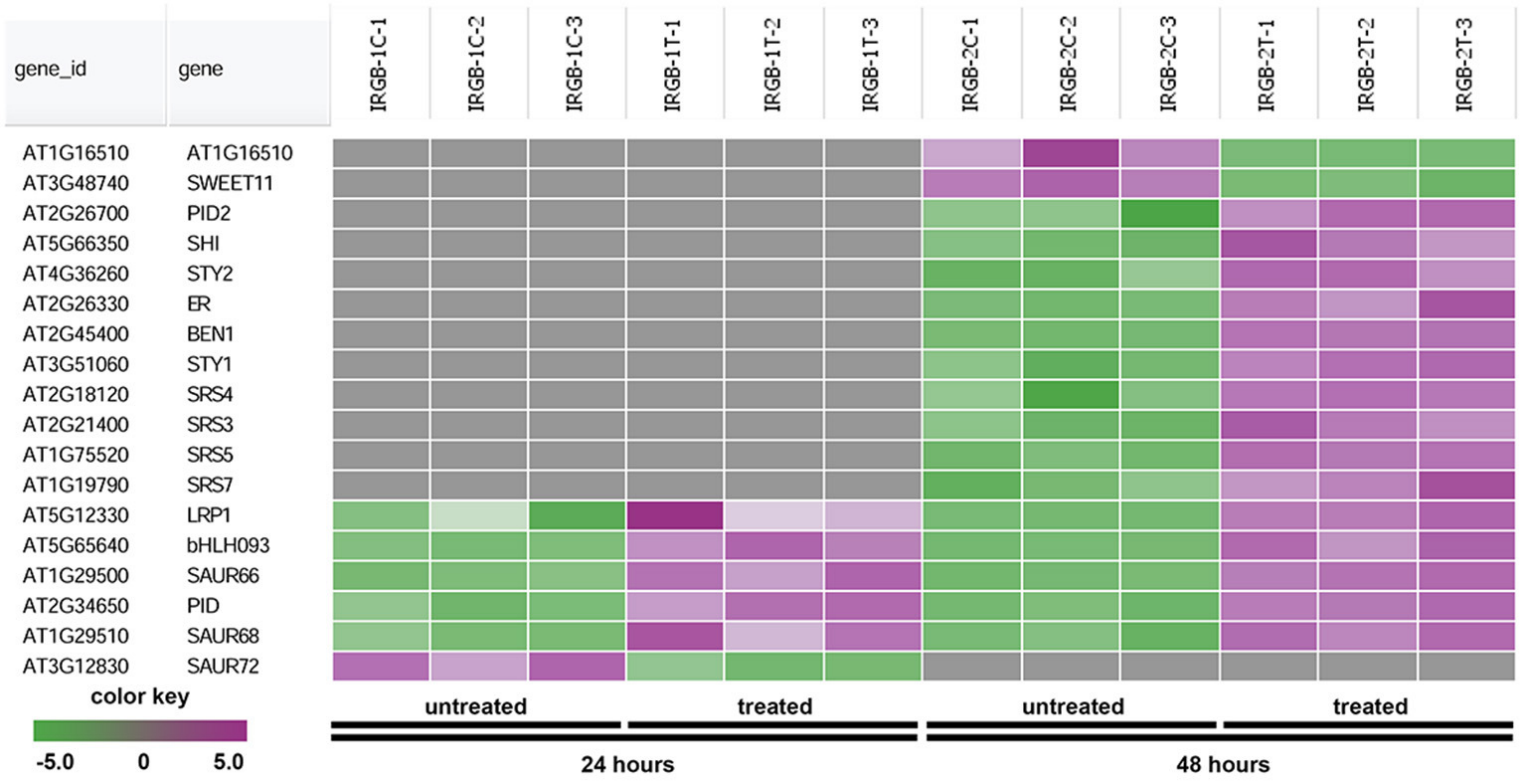
Heatmap showing expression changes of representative genes regulating root growth. The root growth subgroup in the node 2 gene-set showing in Figure 5 consists of a total of 18 genes. Green color, down-regulated; Purple color, up-regulated; and Gray color, no significant expression change.

The heatmap generated from the node 2 found *SHORT INTERNODES* (*SHI*), *SHI-RELATED SEQUENCE* (*SRS/STY*), *PINOID* (*PID*), and *SMALL AUXIN UPREGULATED RNA* (*SAUR*) (Figure 7 and Supplementary Table 4). Genes in the node 2 function in root growth (Figure 7).

**Figure 8:**
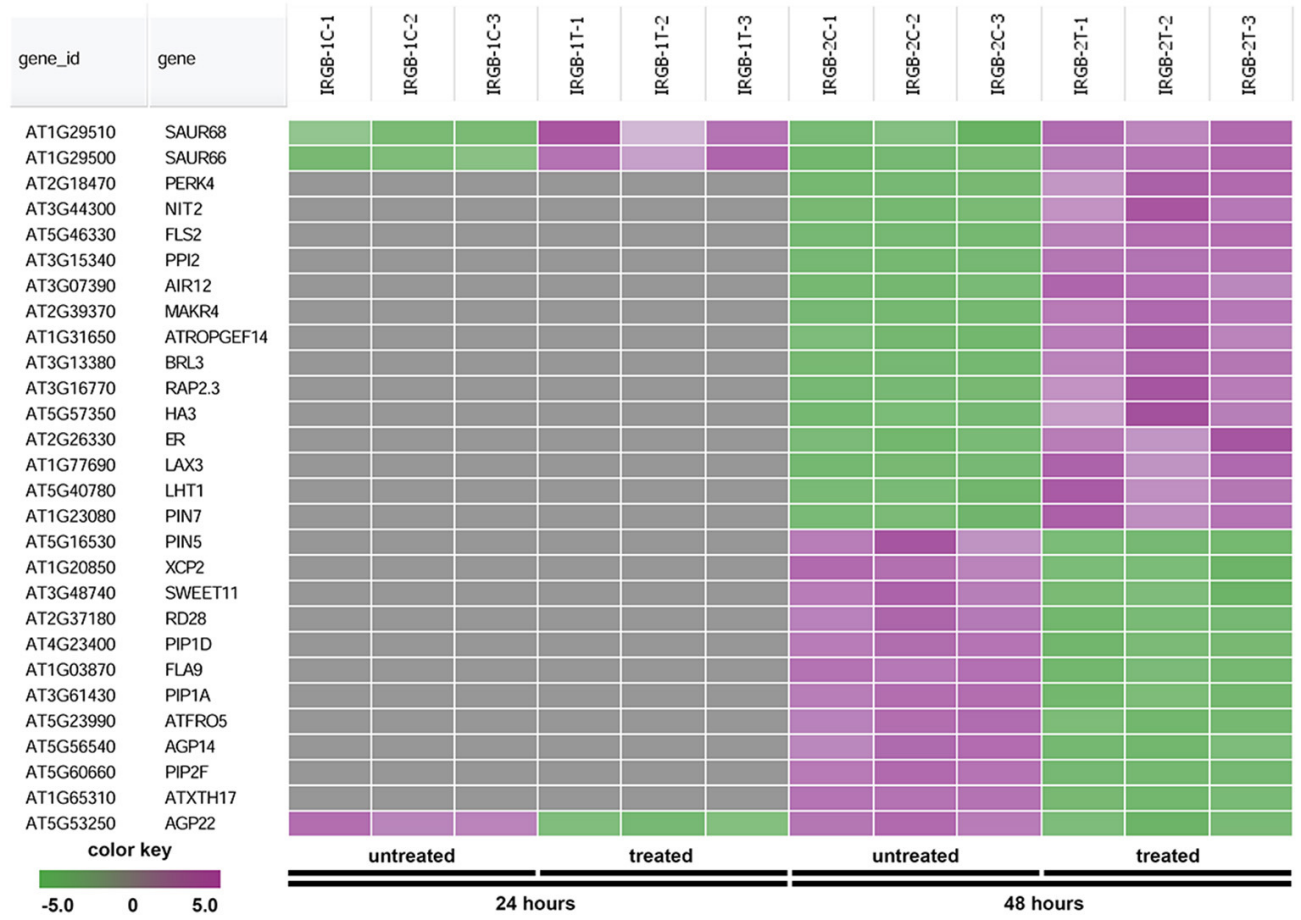
Heatmap showing expression changes of representative genes involved in cell membrane activities. The cell membrane transporter/receptor/water channel subgroup in the node 3 gene-set showing in Figure 5 has a total of 28 genes. Green color, down-regulated; Purple color, up-regulated; and Gray color, no significant expression change.

The node 3 represents the cell membrane transporter/receptor/water channel group which connects to the auxin transport subgroup (Figure 5). The node 3 contains genes encoding receptor kinases (PERK4, BRL3, FLS2, and ER), auxin transporters (PIN5, PIN7, and LAX3), ion transporters/water channels (ATFRO5, PIP1D, RD28, PIP2F, and PIP1A), proton transporters (PPI2 and HA3), sucrose transporters (SWEET11) (Figure 8 and Supplementary Table 5). Those proteins, which are involved in signal transduction, cell wall metabolism and oxidation-reduction process, mostly function in the plasma membrane, cell wall or the extracellular region. In this group, genes were mainly induced at 48 h. Our RNA-seq results suggest that expression of genes mainly involved in auxin signaling is altered in response to colonization by *Rhizobium* sp. IRBG74.

### Expression of *DR5:GUS* is induced by *Rhizobium* sp. IRBG74 treatment

**Figure 9:**
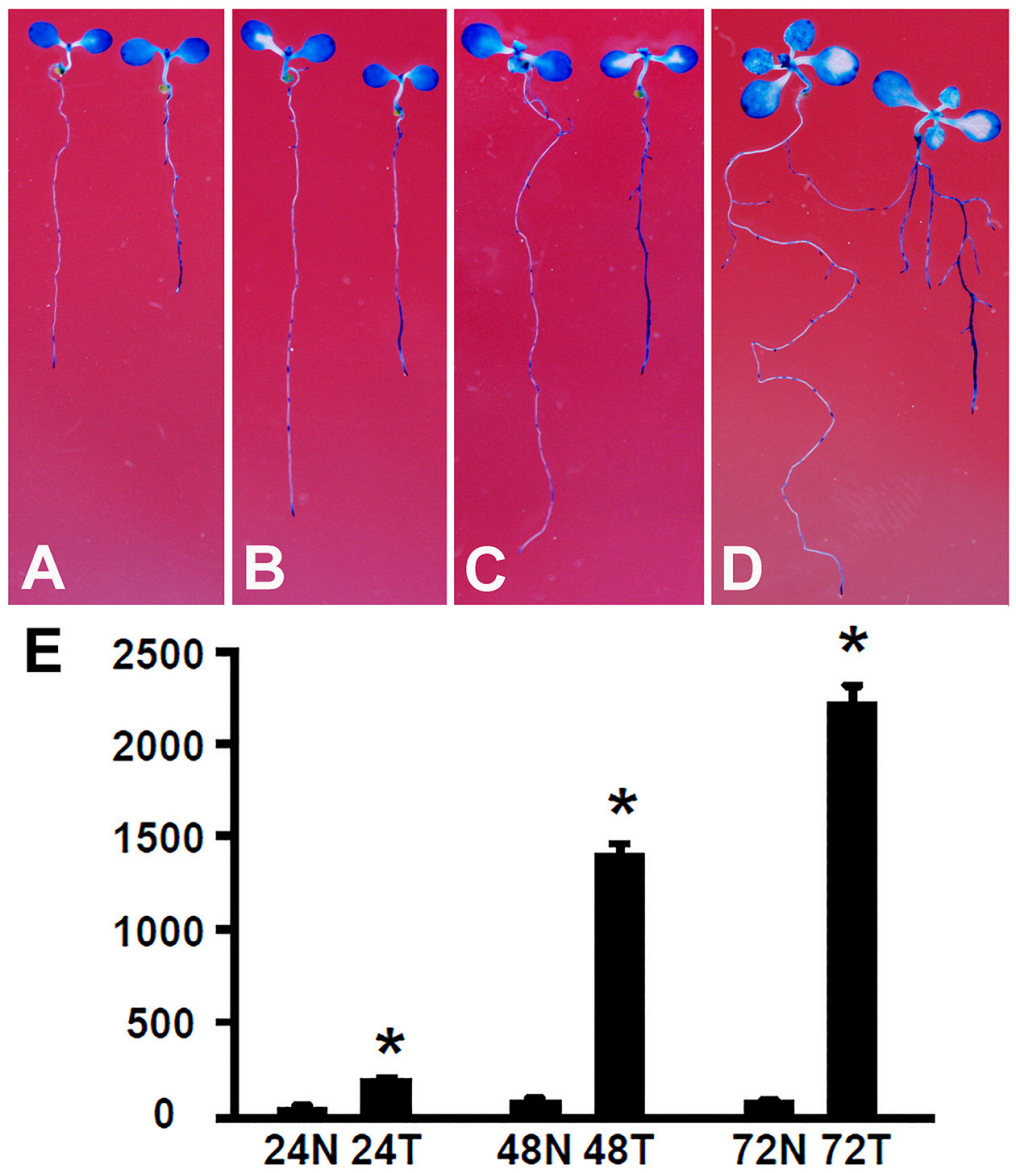
*Rhizobium* sp. IRBG74 affects the *DR5:GUS* expression. **(A–D)** GUS staining showing *DR5:GUS* 4-day seedlings without (left) and with (right) *Rhizobium* sp. IRBG74 treatment for 24 **(A)**, 48 **(B)**, 72 **(C)**, and 120 **(D)** h. **(E)** GUS activity [nM 4-methylumbelliferone (4-MU)/min/mg] quantification of *DR5:GUS* 4-day seedlings without and with *Rhizobium* sp. IRBG74 treatment for 24, 48, and 72 h. 24N: 24 h non-treated, 24T, 24 h treated; 48N, 48 h non-treated; 48T, 48 h treated; 72N, 72 h non-treated, and 72T, 72 h treated. ^*^Indicates the difference is significant (*P* < 0.01).

The root growth phenotype and transcriptome analysis suggest that *Rhizobium* sp. IRBG74 inoculation leads to altered auxin response in *Arabidopsis* roots. To further determine the changes in auxin response, *Arabidopsis* seedlings carrying the auxin response reporter *DR5:GUS* (Ulmasov et al., 1997; Liu et al., 2010) were inoculated with *Rhizobium* sp. IRBG74 and GUS activities were then assayed. After *DR5:GUS* seedlings (30 total, 3 repeats) growing for 24 (Figure 9A), 48 (Figure 9B), 72 (Figure 9C), and 120 (Figure 9D) hours, GUS signals were mainly observed in tips of main and lateral roots without *Rhizobium* sp. IRBG74 treatment; however, enhanced GUS signals were found along main and lateral roots when inoculated with *Rhizobium* sp. IRBG74. Quantitative analysis further showed that GUS activities were significantly increased in roots of *DR5:GUS* seedlings treated by *Rhizobium* sp. IRBG74 for 24, 48, and 72 h (Figure 9E). GUS expression changes were not observed in leaves after *Rhizobium* sp. IRBG74 treatments. Our results support that *Rhizobium* sp. IRBG74 enhances auxin response in *Arabidopsis* roots.

In conclusion, our study shows that the legume nodulating rhizobia *Rhizobium* sp. IRBG74 can colonize roots of the non-legume model plant *Arabidopsis. Rhizobium* sp. IRBG74 enhances lateral root formation but inhibits growth of the main root. Analyses using the auxin perception deficient mutant, auxin response reporter, and RNA-seq suggest that *Rhizobium* sp. IRBG74 affects the main root growth and lateral root formation by altering auxin signaling. Earlier studies have shown that *Rhizobium* sp. IRBG74 synthesizes IAA when supplemented with tryptophan (Biswas et al., 2000a) and its genome (Crook et al., 2013) contains genes that are putatively involved in IAA synthesis. The requirement of supplemented tryptophan for IAA synthesis is a common feature of many plant beneficial bacteria (Idris et al., 2007), including the legume-nodulating rhizobia (Camerini et al., 2008). The biosynthesis of IAA by these bacteria is affected by root exudates (Bais et al., 2006). It has recently been shown that inoculation of cucumber with *Bacillus amyloliquefaciens* results in enhanced secretion of tryptophan by cucumber roots which increases the IAA production by *B. amyloliquefaciens* and thus enhanced plant growth (Liu et al., 2016). In our experiments, the lateral root formation of seedlings which were grown in the basal MS salt media without tryptophan was promoted by *Rhizobium* sp. IRBG74, suggesting that, similar to the cucumber *B. amyloliquefaciens* system, *Rhizobium* sp. IRBG74 likely utilizes tryptophan secreted from *Arabidopsis* roots to synthesize IAA. Further studies are underway to determine whether the observed affects are due to IAA synthesis by *Rhizobium* sp. IRBG74 or due to alteration in plant synthesized IAA upon inoculation with *Rhizobium* sp. IRBG74.

## Author contributions

CZ, DZ, and GP conceived and designed the experiments. DZ and GP supervised the experiments. CZ and JH performed most of the experiments. All authors analyzed data. All authors contributed to writing the article.

### Conflict of interest statement

The authors declare that the research was conducted in the absence of any commercial or financial relationships that could be construed as a potential conflict of interest.
