# Supplementary material for: Rhizobium sp. IRBG74 Alters Arabidopsis Root Development by Affecting Auxin Signaling
Source: Front Microbiol. 2018 Jan 4;8:2556. doi: 10.3389/fmicb.2017.02556 (PMC5759036; doi:10.3389/fmicb.2017.02556)
Supplement: Supplementary file 6 [file Presentation1.PPT]

## Slide 1
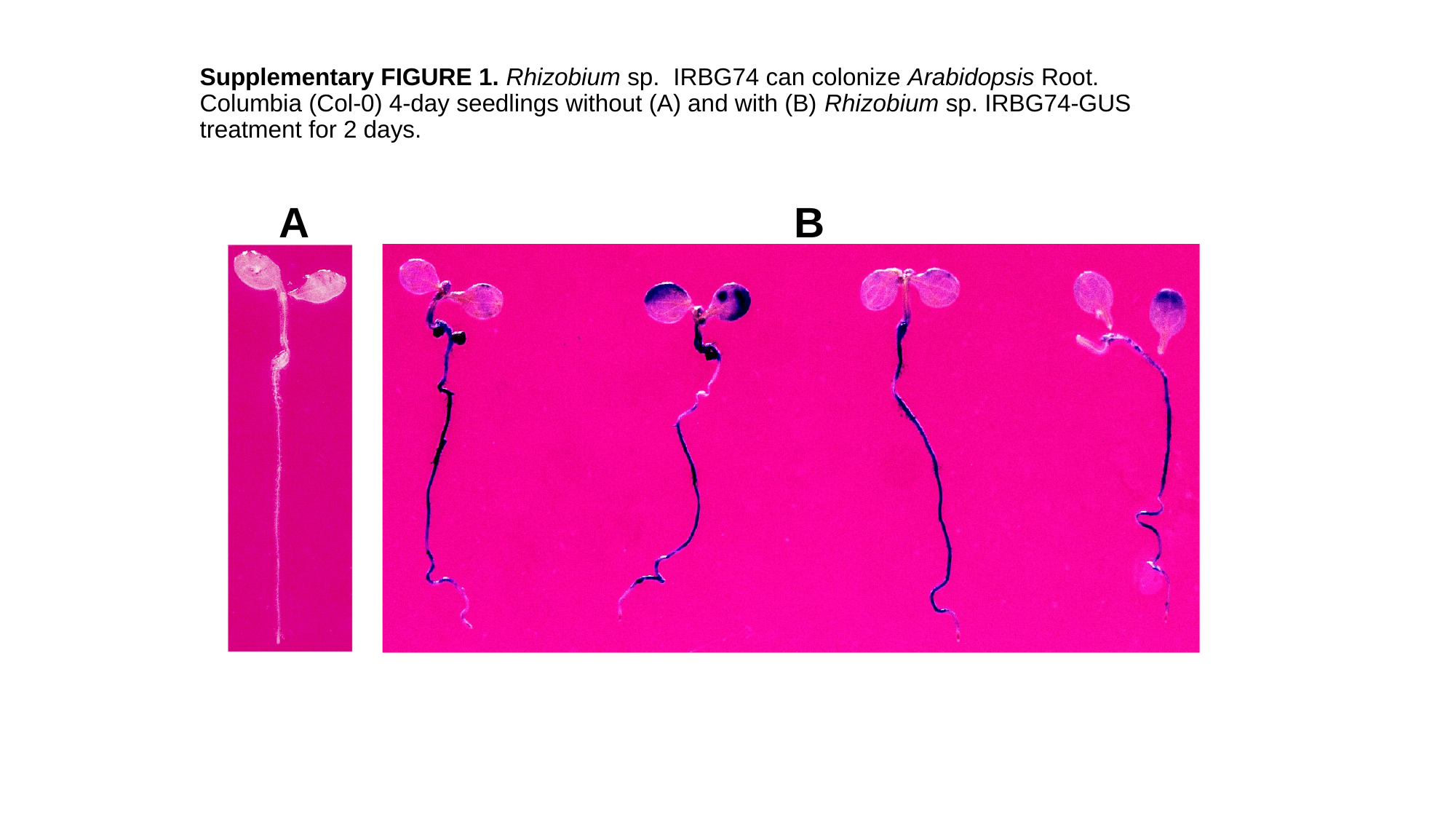

# Supplementary FIGURE 1. Rhizobium sp. IRBG74 can colonize Arabidopsis Root. Columbia (Col-0) 4-day seedlings without (A) and with (B) Rhizobium sp. IRBG74-GUS treatment for 2 days.
A
B

## Slide 2
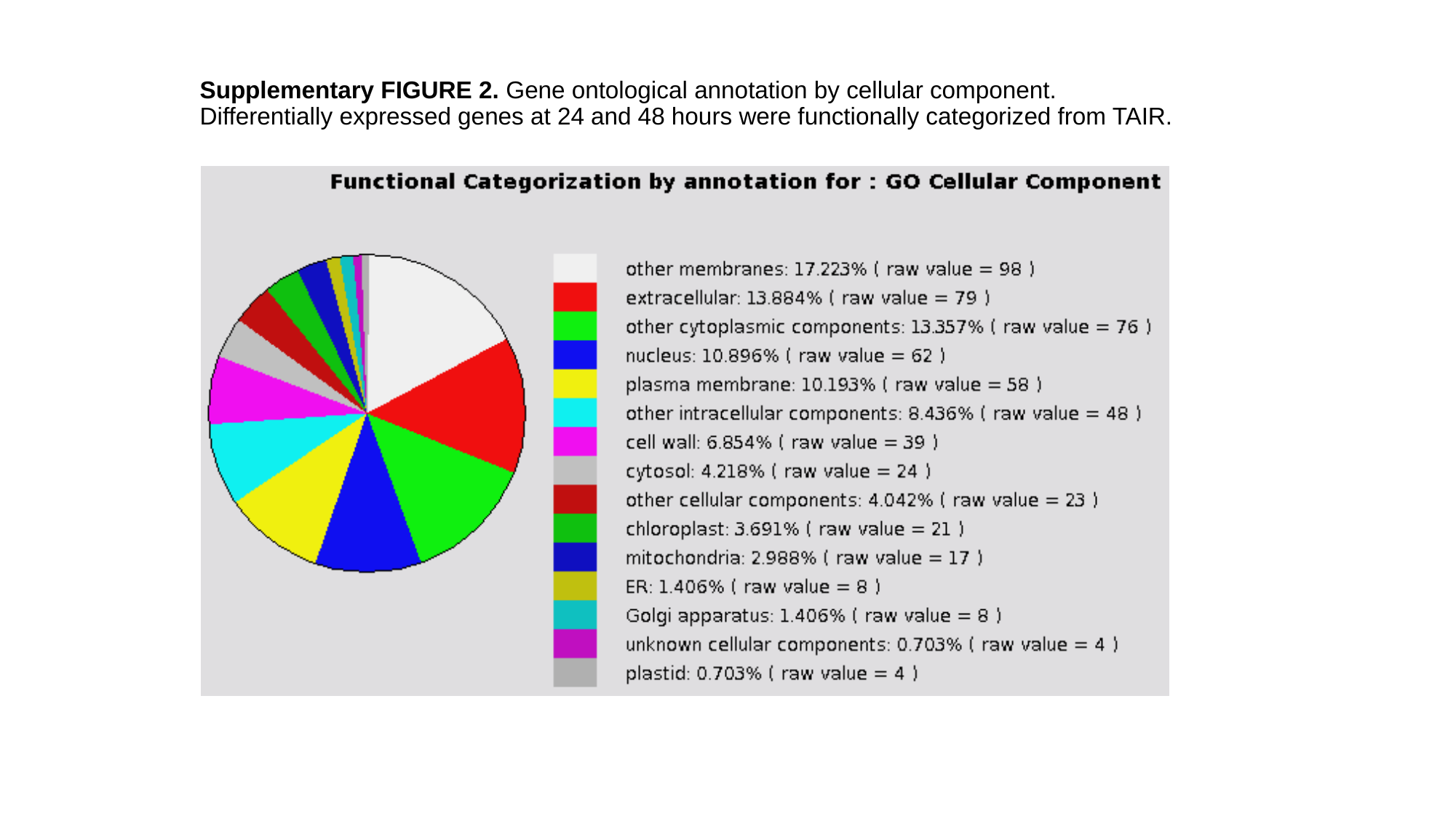

# Supplementary FIGURE 2. Gene ontological annotation by cellular component. Differentially expressed genes at 24 and 48 hours were functionally categorized from TAIR.

## Slide 3
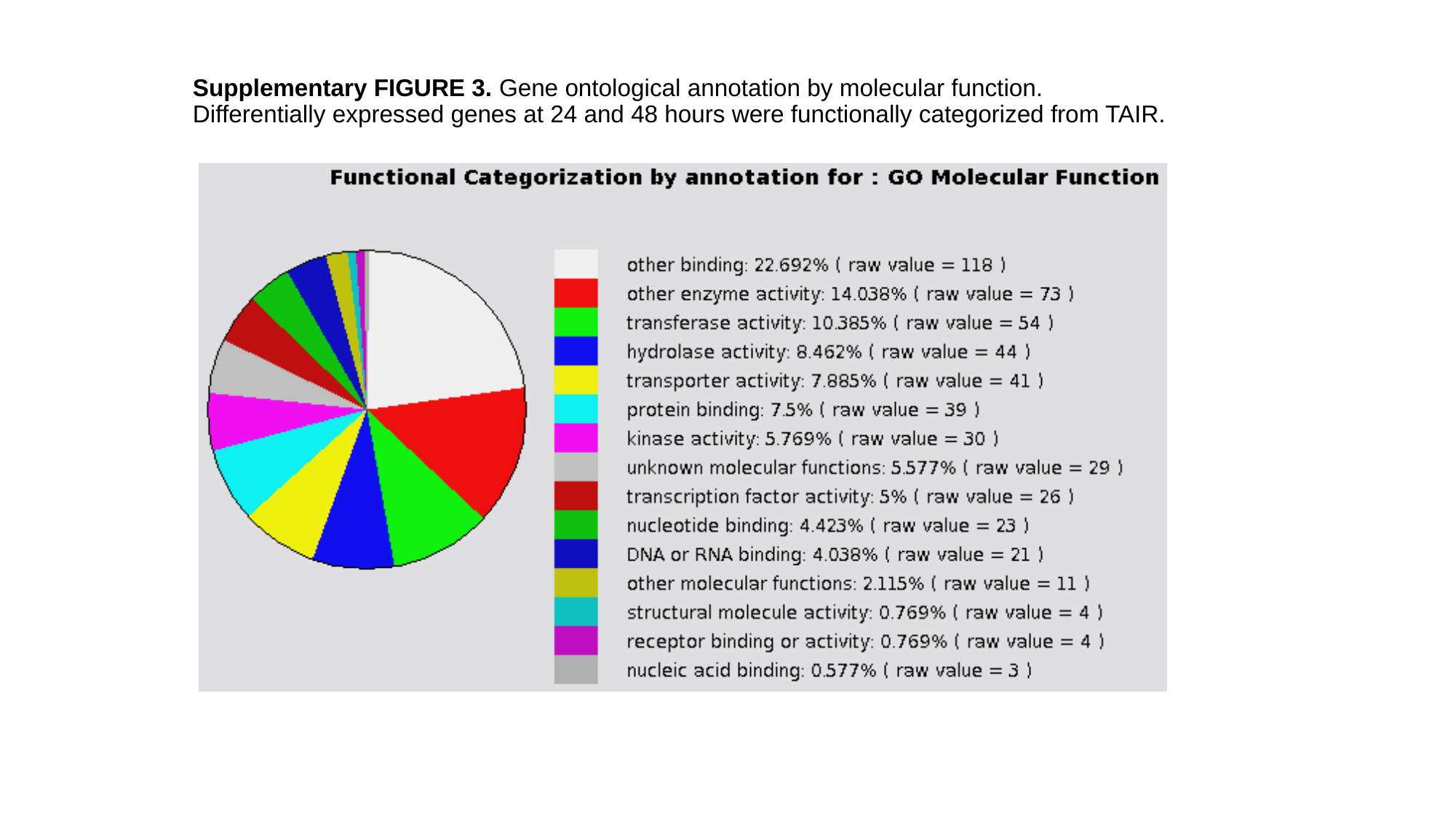

# Supplementary FIGURE 3. Gene ontological annotation by molecular function. Differentially expressed genes at 24 and 48 hours were functionally categorized from TAIR.

## Slide 4
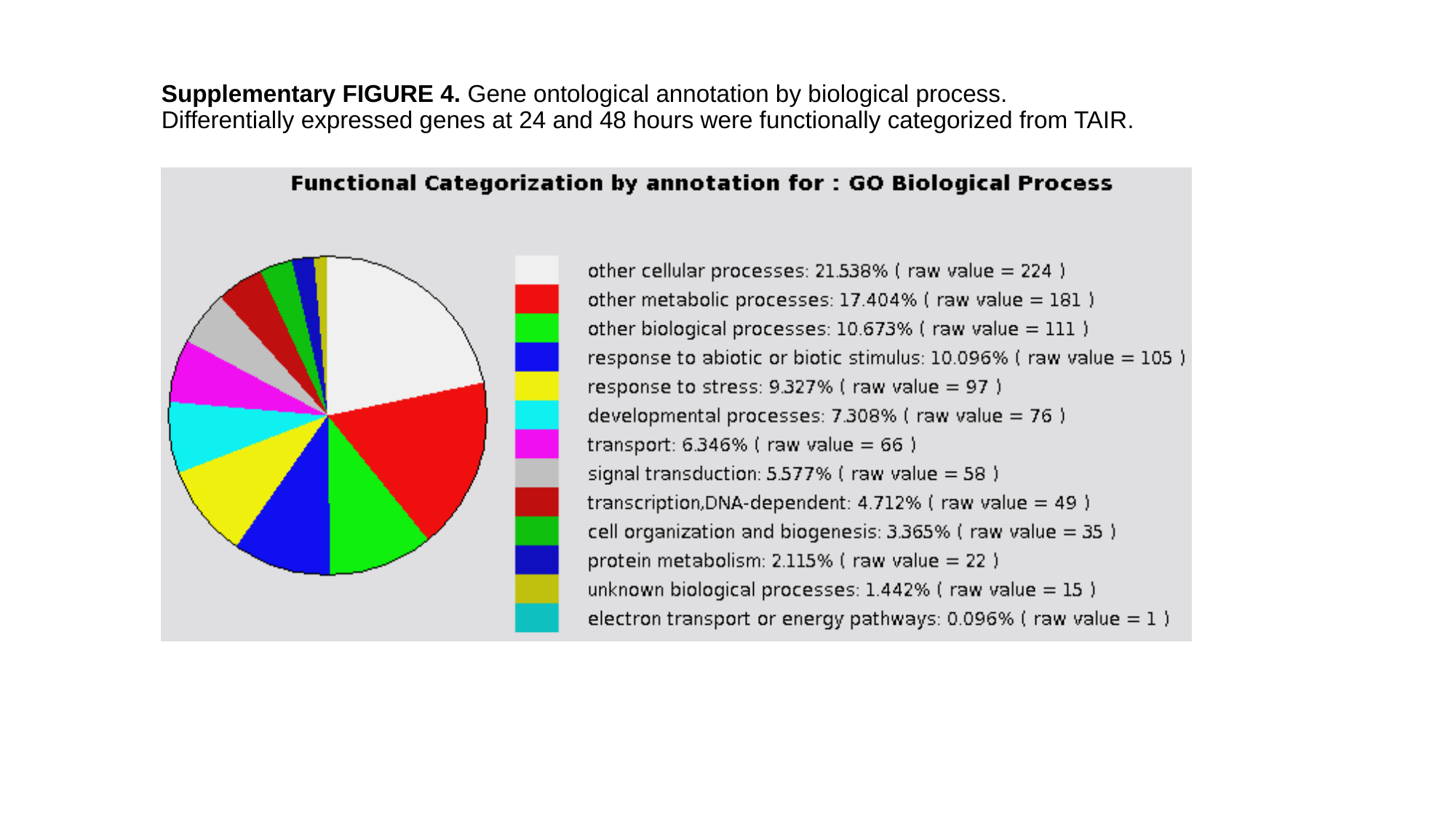

# Supplementary FIGURE 4. Gene ontological annotation by biological process. Differentially expressed genes at 24 and 48 hours were functionally categorized from TAIR.
